# Gastric ulceration causing thoracic spondylodiscitis: a first case of a rare complication post oesophagectomy

**DOI:** 10.1259/bjrcr.20170074

**Published:** 2018-09-01

**Authors:** M Townsend, R Karthigan, H Kaderbhai, C Bailey, D Panchalingam, A Keane, D Nehra, K Burney

**Affiliations:** 1 Epsom and St Helier’s NHS Trust, Carshalton, UK

## Abstract

We report the case of an 84-year-old male, who presented with septicaemia, abdominal and back pain. The patient had a background of oesophageal carcinoma and had undergone previous oesophagectomy and gastric pull-up operation 10 years ago. A computerised topography scan demonstrated a probable gastro-vertebral communication with a destructive process at the T8/T9 vertebral level. Further evaluation with MRI clearly showed the tract between the two structures and confirmed the diagnosis of spondylodiscitis at the adjacent spinal level. The patient was resuscitated, treated with intravenous antibiotics and kept nil by mouth. A subsequent gastroscopy demonstrated an eroding gastric ulcer at the enteric opening of the tract between the tubal stomach and the spinal column. The diagnosis was discussed with the patient, his family and the surgical multidisciplinary team. Given the extent of disease and his multiple medical co-morbidities, the decision was made for conservative management and symptom control. This is the first case of a gastro-vertebral communication causing spondylodiscitis to be described in the literature.

## Clinical presentation

An 85-year-old male presented to the emergency department with tachycardia (110 beats per minute), hypotension (90/50 mmHg) and pyrexia (38.1C). He described a 2-week history of worsening abdominal pain radiating to his back, severely exacerbated by movement. Physical examination demonstrated tenderness in the epigastric region as well as over the lower aspect of the thoracic spine. Biochemically the patient had an elevated leukocyte count and C-reactive protein. He had an extensive medical history including atrial fibrillation, chronic obstructive pulmonary disease, vascular dementia and had also undergone oesophagectomy and gastric pull-up for oesophageal cancer 10 years previously.

**Figure 1.**
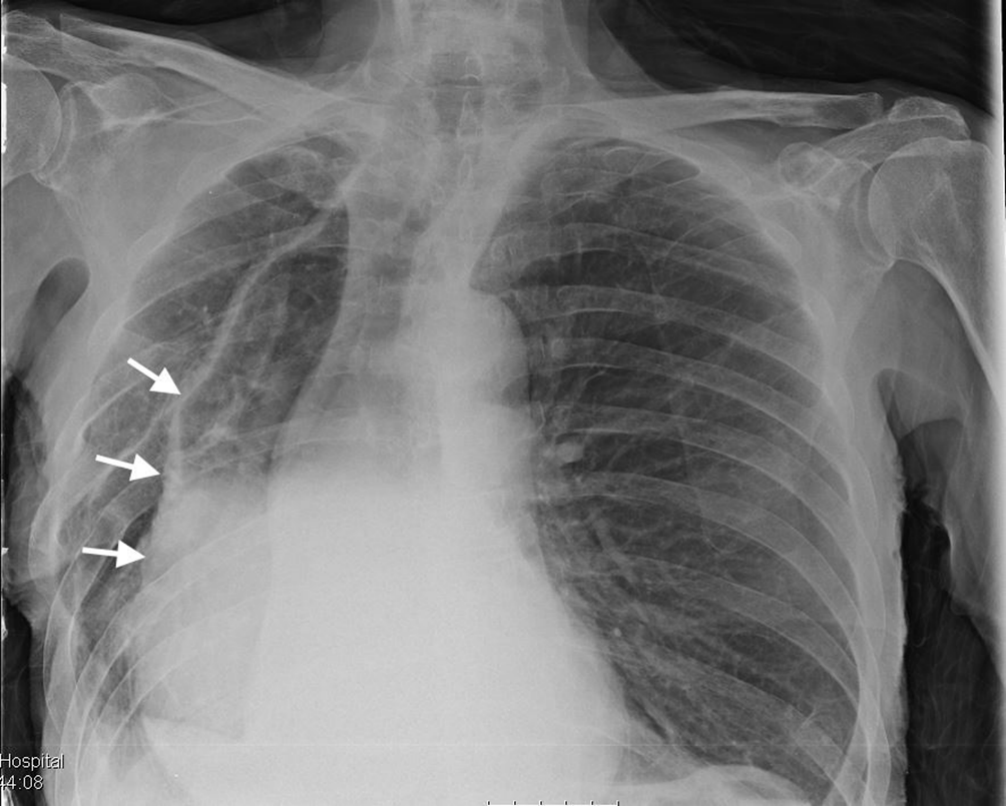
AP erect plain film chest radiograph showing the tubal stomach in the mediastinum following gastric pull-up procedure (arrows). AP, anteroposterior.

**Figure 2.**
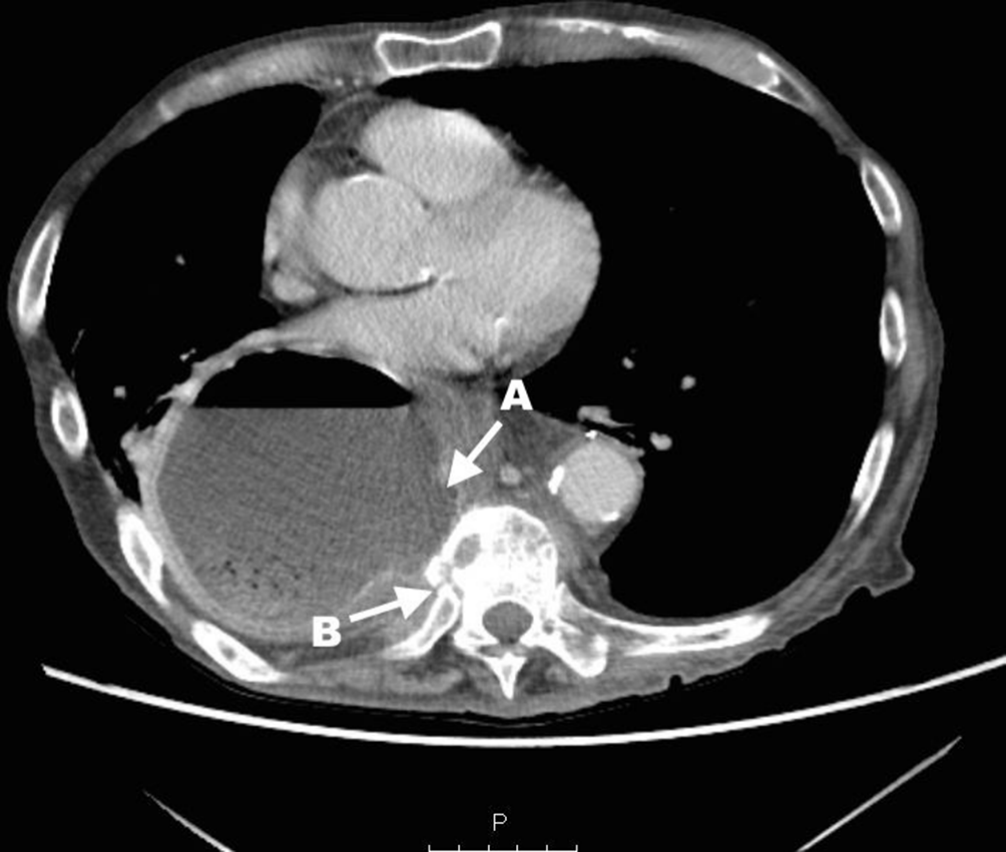
Axial CT image showing a diverticulum arising from the postero medial wall of the stomach (arrow A) which is sited in the mediastinum. The resulting destructive process of the T8 vertebral body can also be seen (arrow B).

**Figure 3.**
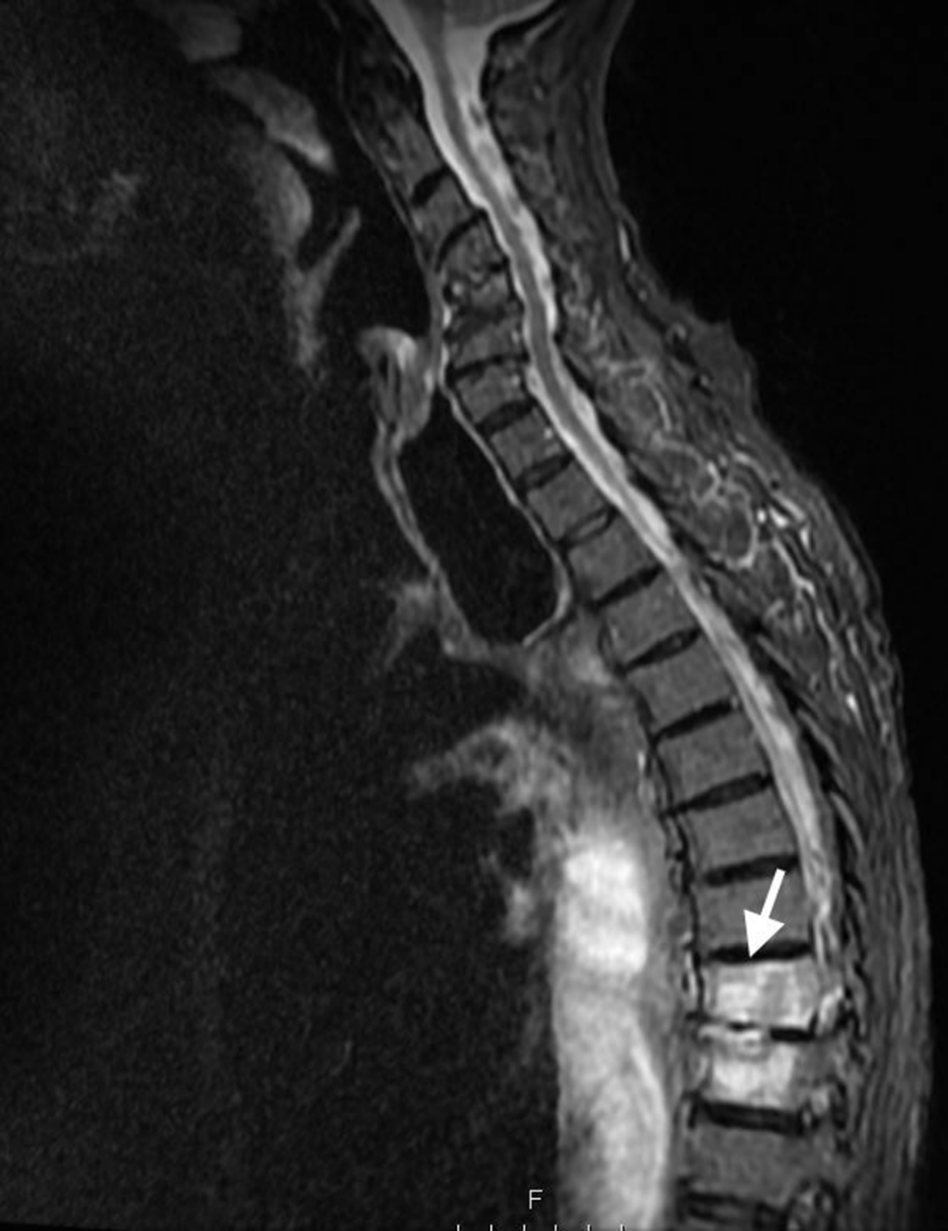
*T*_2_ weighted sagittal MRI image showing enhancement of T8 and T9 vertebral bodies and intervertebral disc consistent with spondylodiscitis.

**Figure 4.**
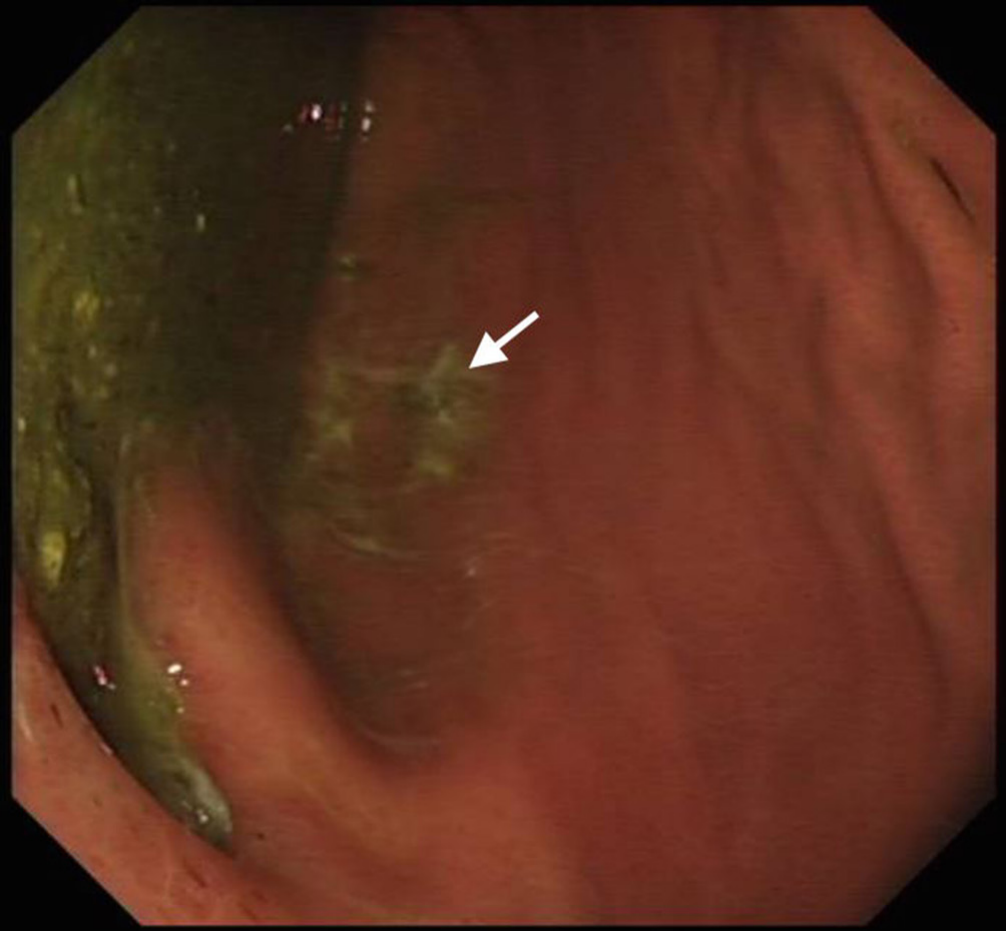
Picture taken at endoscopy which shows an ulceration on the posterior wall of the stomach.

Initial plain film imaging with erect chest X-ray in the emergency department demonstrates this gastric pull-up (Figure 1) Following early resuscitation with oxygen, intravenous fluids and antibiotics, the patient underwent a CT scan. This demonstrated a diverticulum seen arising from the postero medial wall of the pulled stomach with an adjacent aggressive destructive process of the T8 and T9 intervertebral discs (Figure 2) These features were suspicious for a gastro-vertebral communication with resulting spondylodiscitis. A subsequent MRI scan was then performed which helped visualised the tract as well as demonstrating the degree of discitis and osteomyelitis at the T8/T9 level (Figure 3) The communication arising from the tubal stomach was localised, well demarcated and seen only communicating with vertebral column. An OGD was performed to identify the perforation which was due to a gastric ulcer (Figure 4).

## Treatment

Following a multidisciplinary team meeting it was decided that the patient was not a candidate for an operation given his pre-morbid state and extensive medical history. The patient was made nil by mouth and commenced on total parenteral nutrition via a PICC. Intravenous antibiotics were used to treat the spondylodiscitis.

## Outcome, follow-up and discussion

Oesophagectomy and gastric pull-up involves surgical removal of the oesophagus and the interposition of the stomach to re-establish the continuity of the gastrointestinal tract. The procedure is most commonly performed for malignant disease of the oesophagus, as well as some intractable benign conditions such as end-stage achalasia or peptic ulcer disease refractory to medical management.

Oesophagectomy is associated with high rates of perioperative morbidity and mortality. Systemic complications are most often pulmonary in nature and include pneumonia, acute respiratory distress syndrome, exacerbation of pre-existing respiratory conditions and pulmonary embolism. These account for approximately two thirds of post-operative mortality.^1–3^ Common cardiac complications include atrial fibrillation (in up to 20% of patients^4^ and less commonly myocardial infarction (1.1–3.8%).^1,5^

Procedure specific complications of gastric pull-up and oesophagectomy for cancer include anastamotic leak (incidence 5–40%)^3^ stricture (9–40%)^6–8^ conduit ischaemia (9%,^8^ recurrent laryngeal nerve injury (more common with cervical anastomoses and three field lymphadectomy^3,9^ gastritis, necrosis and peptic ulceration of the tubal stomach.^10^ Rarer complications include recurrent cancer of the new tubal stomach, as was in one instance detected first by adenocarcinomatous metastases found in a resected mandibular bone with osteoradionecrosis.^11^ Faecopneumothorax has been reported in a case of post-oesophagectomy herniation and subsequent perforation, of colon into the throrax.^12^ A cardiac tamponade has also been seen post operatively as a result of a communication to a posterior mediastinal chylocele.^13^

There are also those complications which arise from abnormal communications between the tubal stomach and surrounding structures. Broncho-gastric fistulation, is an uncommon but recognised late complication of the operation^14^ and different axial imaging modalities have been suggested to aid in the diagnosis, included the potential benefit of multidetector row CT (MDCT).^15^ A solitary rare case of gastropericardial fistulation has also been described as late sequelae of peptic ulceration following this procedure;^16^ however to the best of our knowledge, no cases of gastro-vertebral communication have previously been reported in the literature.

Spondylodiscitis describes inflammation of the intervertebral discs and the adjacent vertebral bodies. The clinical presentation is often non-specific, and a high index of suspicion is required in combination with suitable imaging modalities to make the diagnosis. Where doubt remains over the diagnosis, a biopsy can be used to confirm the pathology. Spondylodiscitis usually results from haematogenous spread from another site *e.g.* UTI, endocarditis or intravenous drug abuse, but can also arise following iatrogenic inoculation of the area during local injection or surgery. It remains, that in a significant number of cases the primary site of infection is unidentified.^17^ Management includes source identification and treatment with appropriate IV antibiotics. Associated collections or abscesses may need to be drained.

In this case, the tract between stomach and the spinal column makes source control an important consideration. Parenteral nutrition or feeding distal to the site of gastro-vertebral communication (*e.g.* with a nasojejunal tube) should be considered. The use of anti acid medications has the theoretical benefit of reducing acidic secretions to the infection site.

In summary, this case presents the first report of a rare complication of oesophagectomy and gastric pull-up for malignant disease. It highlights the importance of imaging techniques to make the diagnosis of gastro-vertebral communication, and how the application of recognised management principles can be applied to uncommon clinical conditions. With rare presentations such as this, the treatment should be tailored to an individual, taking into consideration pre-morbid function and the wishes of the patient themselves. In this case, the decision was made for conservative management and symptom control during the last days of his life.

## Learning points

- Gastric ulceration of a tubal stomach is a rare but recognised complication of gastric pull-up operation
- This case illustrates the potential for communication between the stomach and any adjacent structures, including soft and bony tissues
- General management including appropriate imaging, nutrition and multidisciplinary team approach to management can be applied to rare and unusual sites enteric communication
